# Perioperative Complications Curriculum for the OB/GYN Resident: A Pilot Study

**DOI:** 10.15694/mep.2019.000193.1

**Published:** 2019-10-22

**Authors:** Allison Staley, AnnaMarie Connolly

**Affiliations:** 1 University of North Carolina

**Keywords:** Curriculum development, surgical complications, perioperative complications, needs assessment, obstetrics and gynecology

## Abstract

This article was migrated. The article was marked as recommended.

**Objectives:** Our objective is to describe a process for development and implementation of a web-based curriculum addressing gynecologic perioperative complications.

**Methods:** Residents, Fellows, and Faculty completed a needs assessment survey addressing satisfaction with baseline perioperative complications curriculum. Residents completed a knowledge pretest. Residents received weekly emails with links to developed topic-specific materials over 4 weeks. Residents completed a post-implementation survey to assess satisfaction and to retest knowledge.

**Results:** 75% (21/28) of Residents and 47% (40/86) Fellows/Faculty completed the needs assessment. 95% (20/21) of Residents and 90% (36/40) Fellows/Faculty reported dissatisfaction with baseline perioperative complications curriculum. Ureteral injury, bowel injury, vaginal cuff dehiscence, and bladder injury were prioritized topics, while assigned readings, interactive web-based case modules, and intraoperative instruction were prioritized modalities for curriculum development. Resident pretest mean score was 72% (40-90%, SD = 15). Weekly curriculum emails were successfully distributed all Resident learners. Eighteen percent of Residents completed the post-implementation survey, with 100% reporting satisfaction with the web-based case modules. Post-test knowledge mean score was 84% (60-100%, SD = 15).

**Conclusion:** Low satisfaction rate with baseline curriculum was reported. Materials were developed and distributed weekly to all Residents, who successfully and consistently accessed the developed web-based curriculum.

## Introduction

The Accreditation Council for Graduate Medical Education (ACGME) mandates compliance with requirements and milestones reporting for Obstetrics and Gynecology (OB/GYN) residency programs. These competency-based requirements and milestones represent a set of baseline expectations to facilitate the development of skills, knowledge, and attitudes in Resident physicians. The milestones were developed through a joint initiative of the ACGME, the American Board of Obstetrics and Gynecology (ABOG), and the American College of Obstetrics and Gynecology (ACOG) in order to provide a framework for curriculum development, addressing competency targets that Residents should be able to perform upon completion of training ( ACMGE Program Requirements for Graduate Medical education in Obstetrics and Gynecology, 2017; The Obstetrics and Gynecology Milestone Project, 2015).

Among these competency targets, Residents should “demonstrate proficiency in perioperative management during their clinical experience.” (program requirement IV.A.6.c) ( ACMGE Program Requirements for Graduate Medical education in Obstetrics and Gynecology, 2017) The required associated milestones include demonstrating the ability to “recognize and manage a surgical complication”; “recognize a perioperative complication”; and, “appropriately utilize an intraoperative consultation.” ( The Obstetrics and Gynecology Milestone Project, 2015) Despite these important procedure-based requirements, there is a paucity of reported curriculum designed to address these areas. Moreover, many intraoperative complications, such as bladder, ureteral, bowel, and vascular injuries, are infrequent and occur in less than 5% of hysterectomy cases, making standardizedexposure unlikely for all trainees in scheduled operative cases ( Stany and Farley, 2008).

As such, our objective is to describe a process for needs assessment, curriculum development, and curriculum implementation of a 4-week web-based perioperative complications program for the OB/GYN Resident at a single institution.

## Methods

**Figure 1.**
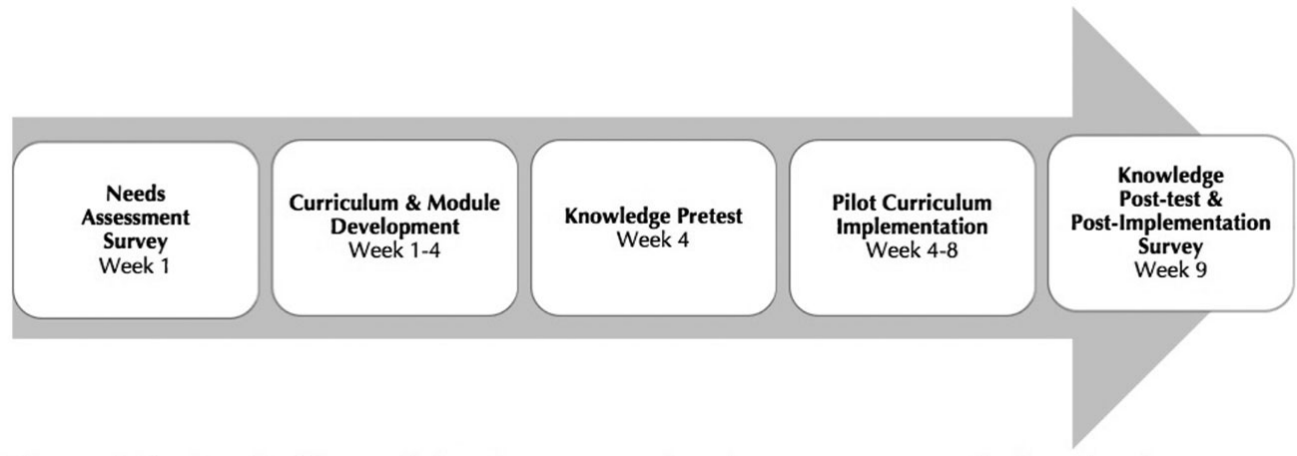
Project timeline outlining the process of needs assessment, curriculum development, knowledge assessment, and curriculum implementation.

This study was granted exemption status (#18-0739) by the Institutional Review Board (IRB) at the University of North Carolina. The project timeline is presented in Figure 1.

### Needs Assessment Survey

Residents, Fellows, and Faculty were sent a Needs Assessment Survey to evaluate satisfaction with the residency program’s baseline perioperative complications curriculum. Respondents were asked to report satisfaction with the program’s baseline curriculum and the ability to address the ACGME perioperative complications milestones, to include teaching Residents how to “recognize and manage a surgical complication”; “recognize a perioperative complication”; and, “appropriately utilize an intraoperative consultation.” Additionally, respondents were invited to prioritize topics for curriculum development and modalities for material delivery in 4-week pilot curriculum. Topic areas proposed included bladder injury, ureteral injury, small bowel injury, wound/vaginal cuff dehiscence, vascular injury, ileus and small bowel obstruction, nerve injury, postoperative fever, and disclosure of a surgical complication. Free text option was also available. Options for curriculum modalities included formal didactic lecture, informal intraoperative discussion, podcast, self-guided web-based case modules, and assigned readings. Free text option was available. Surveys were completed anonymously through Qualtrics® software and were distributed to university email accounts.

### Knowledge Pretest

Prior to distribution of the curriculum, Residents were invited to complete an anonymous, multiple choice 10 question Pre-test on the four prioritized topics identified on the Needs Assessment Survey. The Pre-test was created by the study authors with equal balance of questions across the four prioritized topics. Questions were developed using case-based question stems and, while not formally validated, questions were reviewed by institutional content experts at the study site for clarity and accuracy.

### Pilot Curriculum Development and Implementation

Based on the results of the Needs Assessment Survey, four prioritized topics were identified for the pilot curriculum. The three prioritized modalities for learning material delivery were used to create and deliver the pilot curriculum. Following development of the pilot curriculum, weekly, topic-specific automated emails with links to the curriculum componentswere distributed to Residents through Qualtrics® software over the 4-week curriculum period. This system allowed for tracking of successful distribution and completion of web-based curriculum components.

### Knowledge Post-test

Following completion of the 4-week curriculum, Residents were asked to complete an anonymous Post-test identical to the knowledge Pre-test to assess for interval change in knowledge.

### Post-Implementation Survey

Residents completed an anonymous Post-Implementation Survey to assess satisfaction with the pilot curriculum. If Residents acknowledged not completing specific components of the curriculum, they were asked to describe the barriers to completion. Lastly, Residents were asked to rate the efficiency and ease of access to curriculum resources through our web-based distribution.

Statistical analysis was performed using SPSS version 19.0 (IBM Corp, Chicago, IL). Differences in data are described with appropriate descriptive statistics. The Needs Assessment Survey and Post-Implementation Survey satisfaction data are presented with responses of “strongly agree” and “agree” considered as “satisfied” and responses of “neutral,” “disagree,” and “strongly disagree” considered as “unsatisfied”.

## Results/Analysis

### Needs Assessment Survey

Of the 114 surveys distributed, 75% (21/28) of Residents and 47% (40/86) of Fellows/Faculty responded to the Needs Assessment Survey, as demonstrated in Table 1. Fellow/Faculty respondents included 42.5% (17/40) specialists in general Obstetrics and Gynecology (OBG); 28% (11/40) Gynecologic Oncology (GYN ONC); 15% (6/40) Maternal Fetal Medicine (MFM); 7.5% (3/40) Minimally Invasive Gynecology Surgery (MIGS); 5% (2/40) Female Pelvic Medicine and Reconstructive Surgery (FPMRS); and 2.5% (1/40) Reproductive Endocrinology and Infertility (REI).

**Table 1.** Needs Assessment Survey completion rates and reported satisfaction with baseline perioperative complication curriculum from Resident, Faculty, and Fellow physician respondents.

Survey Completion Rate	
Residents	21/28 (75%)
Faculty/Fellows	40/86 (47%)
Specialists in General Obstetrics and Gynecology	17/40 (42.5%)
Gynecologic Oncology	11/40 (28%)
Maternal Fetal Medicine	6/40 (15%)
Minimally Invasive Gynecology Surgery	3/40 (7.5%)
Female Pelvic Medicine and Reconstructive Surgery	2/40 (5%)
Reproductive Endocrinology and Infertility	1/40 (2.5%)
**Satisfaction with Baseline Curriculum**	
Residents	1/21 (5%)
Faculty/Fellows	4/40 (10%)

**Figure 2.**
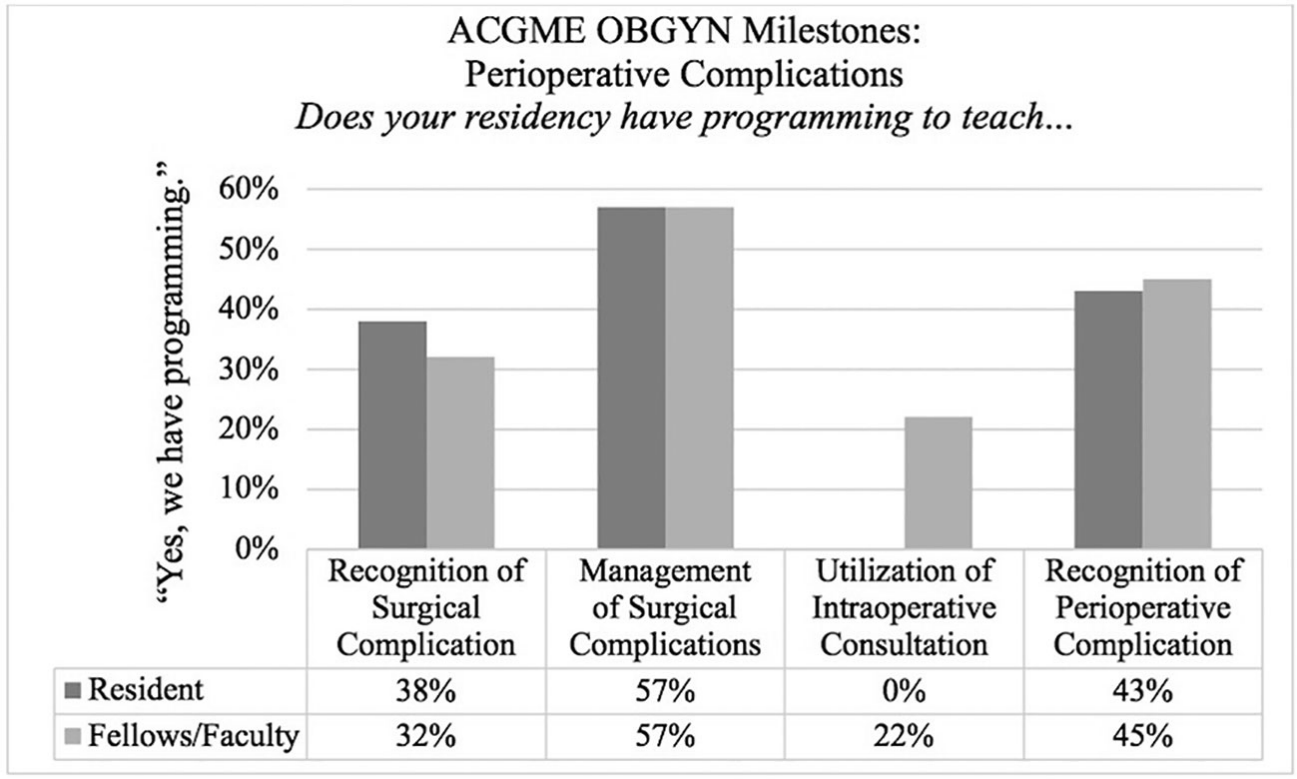
Residents, Fellows, and Faculty report Residency has programming to teach ACGME OB/GYN Perioperative Milestones (from milestone sets of *Perioperative Complications, GYN Technical Skills–Endoscopy, Laparotomy, & Vaginal Surgery*)

Satisfaction with the residency program’s baseline perioperative complications curriculum was quite low with only 5% (1/21) of Residents and 10% (4/40) of the Fellow/Faculty respondents reporting satisfaction. As seen in Figure 2, 38% (8/21) of Residents and 32% (13/40) of Fellows/Faculty reported that programming was in place to teach recognition of surgical complications; 57% (12/21) of Residents and 57% (23/40) of Fellows/Faculty reported programmingwas in place to teach management of surgical complications; none (0/21) of the Residents and 22% (9/40) of Fellows/Faculty reported programming was in place to teach utilization of appropriate intraoperative consultations; and, 43% (9/21) of Residents and 45% (18/40) of Fellows/Faculty reported programming to teach recognition of a perioperative complication.

When asked to prioritize topics for pilot curriculum inclusion, respondents rank ordered topics from higher to lower priority, as follows: bladder injury, ureteral injury, small bowel injury, and wound/vaginal cuff dehiscence, respectively. When asked to prioritize modalities for curriculum delivery, informal intraoperative instruction, web-based case module instruction, and assigned readings were given highest priority, respectively.

### Pilot Curriculum Development

The curriculum was then developed for the web-based 4-week program addressing the prioritized topics of bladder injury, ureteral injury, small bowel injury, and wound/vaginal cuff dehiscence. The study authors utilized the three prioritized modalities for learning material delivery, which included intraoperative instruction, web-based module instruction, and assigned readings. Web-based case modules were developed using ABOG, ACGME, and Council on Resident Education in Obstetrics and Gynecology (CREOG) requirements and objectives with topic-specific materials including text, images, and embedded case-based self-assessment questions. Weekly automated emails were sent to all Residents. Links to the week’s topic-specific materials, including web-based case module, 1-2 assigned readings, and a reminder to participate in an informal intraoperative discussion on the topic. Faculty and Fellows were also provided weekly emails to encourage participation in informal intraoperative topic-specific discussion.

### Pilot Curriculum Implementation

The weekly curriculum emails were successfully delivered via Qualtrics® automated messaging. The web-based distribution success was 100%, with all 4 weekly emails delivered to 28 residents over 112 total messages.

### Post-Implementation Survey Results

Post-Implementation Surveys were sent to all Residents following completion of the 4-week curriculum. There were five Resident respondents. Regarding the web-based case-based modules, 100% of these respondents reported satisfaction with each of the four modules. Qualtrics® programming was queried to identify number of residents who viewed the weekly web-based case-based modules as well as the number who successfully completed the entire learning module. As seen in Table 2, of the Residents who accessed the modules, 100% fully completed the modules. Fifty percent (14/28) of Residents completed the ureteral injury web-based module (Week #1); 36% (10/28) completed the small bowel injury web-based module (Week #2); 29% (8/28) completed the wound/vaginal cuff dehiscence web-based module (Week #3); and, 18% (5/28) completed the bladder injury web-based module (Week #4).

Utilization of the weekly assigned readings was assessed on the Post-Implementation Surveys. Of the Resident respondents, 80% (4/5) completed Week #1 readings; 40% (2/5) for Week #2; 40% (2/5) for Week #3; and, 40% (2/5) for Week #4. All respondents who did complete the readings endorsed 100% satisfaction with the reading assignments. When asked to identify barriers to completing the reading assignment, all respondents reported time availability as the major barrier.

**Table 2.** Access and completion rate of weekly online case-based module component as recorded by Qualtrics®. Of the Residents who accessed the module, 100% completed the module case successfully.

Module	Access & Completion Rate
Ureteral Injury	14/28 (50%)
Small Bowel Injury	10/28 (36%)
Vaginal Cuff Dehiscence	8/28 (29%)
Bladder Injury	5/28 (18%)

Respondent completion of topic-specific intraoperative discussion with a Faculty or Fellow was also queried. Among the respondents, intraoperative discussions occurred with only 20-40% of respondents during the 4-week pilot curriculum. The barriers to completion of intraoperative teaching included no intraoperative experience that week, the attending or Fellow did not initiate a teaching opportunity, or the Resident did not initiate a teaching opportunity. Lastly, 100% (5/5) of Residents reported that the weekly emails made resources easier to access and more efficient. Only 20% (1/5) reported that the weekly prompts to encourage intraoperative discussion were effective.

### Knowledge Pretest & Post-test Scores

The knowledge Pre-test was distributed successfully to all Residents prior to curriculum implementation. Fifty-seven percent (16/28) of Residents completed the Pre-test, with a mean score of 72% (40-90%, SD = 15). The knowledge Post-test was completed by 18% (5/28) of Residents. All Residents who completed the Post-test previously completed the Pre-test. The Post-test mean score was 84% (60-100%, SD = 15), demonstrating no significant difference between the two groups ( *p* = 0.11).

## Discussion

The gynecologic surgeon must be prepared to identify and manage associated perioperative complications. The ACGME program requirements and milestones outline components of perioperative complication management as graduation targets for Resident physicians. Yet, standardizing clinical training for all Resident physicians proves challenging due to the infrequency of many perioperative complications. Results from our institution’s Needs Assessment Survey identified poor satisfaction with our residency program’s current perioperative complications curriculum, noting limited programming to address each of the four ACGME milestones. As such, we report a process for assessing program needs, creation of a pilot curriculum, and evaluation of the implemented pilot curriculum.

Our program prioritized the 4 topic areas of bladder injury, ureteral injury, small bowel injury, and wound/vaginal cuff dehiscence for inclusion in our 4-week pilot curriculum. Our program then prioritized the modalities for curriculum delivery to include self-directed web-based case modules, assigned subject-specific readings, and reminders to Residents and Faculty/Fellow teachers to perform topic-specific weekly intraoperative teaching. Distribution of the curriculum emails was successful with automated messages sent to every Resident each week of the curriculum. By query of the Qualtricsâ software, we identified 20-50% of all Residents had completed the web-based case modules each week, with excellent satisfaction scores on the Post-Implementation Survey. Completion of the assigned readings and intraoperative instruction was low among those who responded to the Post-Implementation Survey. Notably, direct comparison of the three curriculum modalities is challenging as Qualitrics® software was used to track web-based module completion, and Resident self-report on the Post-Implementation Survey was required to assess completion of assigned readings and intraoperative discussion. Among the reported barriers to assignments were limited time and lack of initiation of intraoperative discussion by the Faculty or Resident.

Changes in the clinical learning environment, such as restricted trainee duty hours, increased supervision with decreased trainee autonomy, and a more stringent medicolegal environment, have markedly altered how graduate medical education is conducted ( Nasca, Day, and Amis, 2010; Philibert, Friedmann, and Williams, 2002). Such changes have raised concerns for graduate preparedness for independent practice, particularly in procedure-based specialties ( Espey, Ogburn, and Puscheck, 2007; Feanny *et al.*, 2005; Carlin, Gasevic, and Shepard, 2007). A 2015 survey of 218 fellowship directors of ABOG-accredited programs reported on these concerns in Obstetrics and Gynecology Residents entering fellowship training. Roughly 20-50% of MFM, REI, GYN ONC and FPMRS directors reported that current or past fellows could not recognize a surgical complication on entry to fellowship. Reported potential contributors to this finding included decreased Resident trainee autonomy in the operating room; surgical volume exposure during Residency training in obstetric versus gynecologic subspecialties; and, varying expectations for technical skill and surgical performance among the different subspecialties ( Guntupalli *et al.*, 2015). While milestones assessment may help, curriculum directly focused on procedural skill development, including the handling of perioperative complications, may address inconsistencies in trainee experience and education in gynecologic surgery and perioperative care. The increased clinical demands of learners and teachers, as well as limited available time, highlight the importance of efficient, accessible learning tools in graduate medical education. Our pilot curriculum was intentionally designed to address these concerns.

Curriculum delivery via web-based materials may be an efficient and effective means of education for adult learners with certain advantages over standard print materials. Among these advantages include the ability to provide interactive teaching with a case-based application. In a randomized trial of a web-based learning tool versus standard printed material, Internal Medicine Residents attained similar post-assessment knowledge scores between the two modalities; however, those who used web-based learning spent less time reviewing the provided material ( Bell, Fonarow, Hays, and Mangione, 2000). Furthermore, the ability to incorporate embedded self-assessment questions actively engages learners and has been shown to improve learning outcomes for Resident trainees ( Cook *et al.*, 2006). Additional advantages of the web-based format include quick performance feedback, completion tracking for trainees and instructors, as well as the convenience to complete the learning activity regardless of time or location ( McKimm, Jollie, and Cantillon, 2003).

In summary, we demonstrate a process for development and implementation of a web-based curriculum for management of perioperative complications. Our pilot curriculum implementation demonstrates that web-based learning is accessible to and desired by Residents trainees. Given the relative infrequency of perioperative complications, intentional programming addressed towards this important subject area seems reasonable, and a multi-institutional study implementing this pilot program is currently underway.

## Conclusion

Low satisfaction rate with baseline perioperative complications curriculum was reported. Materials were developed based on needs assessment survey results. The multi-modal curriculum was distributed weekly to all Residents, who successfully and consistently accessed the developed web-based curriculum.Following implementation of the 4-week pilot curriculum, a trend in improve knowledge was identified on post-test scores. Residents reported high satisfaction with the developed web-based curriculum, particularly online case-based modules.

## Take Home Messages

• Obstetrics and Gynecology Residents report poor satisfaction with perioperative complications curriculum in gynecologic surgery
• Residents report limited curriculum available to address the ACGME knowledge requirements for managment of perioperative complications
• Residents report bladder injury, ureteral injury, small bowel injury, and vaginal cuff/wound dehiscence as the most highly priortized topics for curriculum developement
• Residents report high satisfaction with a web-based curriculum, particularly interactive online case-based modules
• Resident knowledge improved following implementation of a 4-week web-based perioperative complications curriculum

## Notes On Contributors

Dr. Allison Staley is a Gynecologic Oncology Fellow in the Department of Obstetrics and Gynecology at the University of North Carolina at Chapel Hill.

Dr. AnnaMarie Connolly is a Professor of Urogynecology and Reconstructive Pelvic Surgery, the Vice Chair for Education, Director of the Medical Education Division, and Residency Director in the Deparment of Obstetrics and Gynecology at the University of North Carolina at Chapel Hill.

## References

[ref1] ACGME Program Requirements for Graduate Medical Education in Obstetrics and Gynecology . (2017) Obstet Gynecol. Accredit Counc Grad Med Educ.

[ref2] Bell D.S. Fonarow G.C. Hays R.D. and Mangione C.M. (2000) Self-study from web- based and printed guideline materials: a randomized, controlled trial among resident physicians. Ann Intern Med. 132, pp.938–946. 10.7326/0003-4819-132-12-200006200-00003 10858176

[ref3] Carlin A.M. Gasevic E. and Shepard A.D. (2007) Effect of the 80-hour work week on resident operative experience in general surgery. Am J Surg. 193, pp.326–329. 10.1016/j.amjsurg.2006.09.014 17320528

[ref4] Cook D. Thompson W.G. Thomas K.G. Thomas M.R. (2006) Impact of self-assessment questions and learning styles in Web-based learning: a randomized, controlled, crossover trial. Acad Med. 81(3), pp.231–238. 10.1097/00001888-200603000-00005 16501263

[ref5] Espey E. Ogburn T. and Puscheck E. (2007) Impact of duty hour limitations on resident and student education in obstetrics and gynecology. J Reprod Med. 52, pp.345–348. 17583230

[ref6] Feanny M.A. Scott B.G. Mattox K.L. and Hirshberg A. (2005) Impact of the 80-hour work week on resident emergency operative experience. Am J Surg. 190, pp.947–949. 10.1016/j.amjsurg.2005.08.025 16307951

[ref7] Guntupalli S.R. Doo D.W. Guy M. Sheeder J. (2015) Preparedness of obstetrics and gynecology residents for fellowship training. Obstet Gynecol. 126, pp.559–568. 10.1097/AOG.000000000000099 26244537

[ref8] McKimm J. Jollie C. and Cantillon P. (2003) ABC of learning and teaching: Web based learning. BMJ. 326(7394), pp.870–873. 10.1136/bmj.326.7394.870 12702625 PMC1125774

[ref9] Nasca T.J. Day S.H. and Amis ES Jr. (2010) ACGME Duty Hour Task Force. The new recommendations on duty hours from the ACGME Task Force. N Engl J Med. E3, pp.363. 10.1056/NEJMsb1005800 20573917

[ref10] Philibert I. Friedmann P. Williams W.T. (2002) ACGME Work Group on Resident Duty Hours, Accreditation Council for Graduate Medical Education. New requirements for resident duty hours. JAMA. 288, pp.1112–1114. 10.1001/jama.288.9.1112 12204081

[ref11] Stany M.P. and Farley J.H. (2008) Complications of Gynecologic Surgery. Surg Clin N Am. 88, pp.343–359. 10.1016/j.suc.2007.12.004. 18381117

[ref12] The Obstetrics and Gynecology Milestone Project . (2015) The Accreditation Council for Graduate Medical Education, The American Board of Obstetrics and Gynecology, and The American College of Obstetrics and Gynecology. https://www.acgme.org/Portals/0/PDFs/Milestones/ObstetricsandGynecologyMilestones.pdf( Accessed: 04-12-19)

