# Supplementary material for: Perioperative Complications Curriculum for the OB/GYN Resident: A Pilot Study
Source: MedEdPublish (2016). 2019 Oct 22;8:193. [Version 1] doi: 10.15694/mep.2019.000193.1 (PMC10712462; doi:10.15694/mep.2019.000193.1)
Supplement: Peer review history

# Reviewer report: Reviewer response for version 1

**DOI:** 10.21956/mep.19891.r30099

**Published:** 2020-02-04

**Authors:** Ken Masters

**Affiliations:** 1 Sultan Qaboos University

This review has been migrated. The reviewer awarded 3 stars out of 5 An interesting paper on the development of a web-based Ob/Gyn Resident training intervention. The research was performed in a simple no-frills, logical and effective manner: Needs assessment, pre-test, intervention development, intervention delivery, post-test, data analysis. This is pretty much a solid way of doing things, and the authors have performed it well.It is unfortunate that only 5 Residents responded to the survey and post-test. Although one can give percentages, when the number is so low, percentages and significant similarities and differences are difficult to interpret. This is especially so because post-surveys can be biased in favour of successful or positive candidates. I think the line in the conclusion “a trend in improve[d] knowledge was identified on post-test scores” might not be accurate, given that the Result state: “The Post-test mean score was 84% (60-100%, SD = 15), demonstrating no significant difference between the two groups (p = 0.11).”It also would have been useful if some more detail about the course (perhaps a supplementary file with screenshots (copyright permitting), and possibly a link to it, if publicly available) could be given to the reader. Minor issue: “was quite low” could probably be changed to “was low”.Overall, a useful Pilot of a concept; the low response rates in the post-intervention survey and test, however, are problematic. While they certainly do not indicate a negative trend, the strength of the results is questionable. If the researchers are confident in their work, though, I look forward to seeing a repeat of the research in which the conclusions are more soundly supported.

Reviewer Expertise:

NA

No decision status is available

# Reviewer report: Reviewer response for version 1

**DOI:** 10.21956/mep.19891.r30098

**Published:** 2019-10-23

**Authors:** BALAJI ARUMUGAM

**Affiliations:** 1 TAGORE MEDICAL COLLEGE AND HOSPITAL

This review has been migrated. The reviewer awarded 4 stars out of 5 Dear Allison Staley and Anna,Kudos to both of you for publishing such a nice neat work on implementation of a web based curriculum for the residents and faculty. The best part of the curriculum development and implementation in this study is needs assessment survey, before the initiation of the study. Its surprising that the results of the needs assessment survey results showed that the majority reported dissatisfaction with baseline perioperative complications curriculum. This survey has given the guidelines to focus on the aspects of the perioperative complications curriculum on Ureteral injury, bowel injury, vaginal cuff dehiscence, and bladder injury were prioritized topics. The pre and post curriculum implementation scores diffidence was not huge and it showed only 12% improvement in scores. But the satisfaction rate on web based modules and the curriculum was 100% reporting satisfaction. Anyways developing a web based modules on perioperative complications curriculum in a subject specialty is not easy, but it was done clearly by this duo. Keep working on it more and improve the curriculum in such a way that the post test scores reaching maximum compared to the pre test scores. Suggestions: Instead of MCQs - some other mode of assessment could have been better to assess the pre and post test scores.

Reviewer Expertise:

NA

No decision status is available
